# Improved Processability and the Processing-Structure-Properties Relationship of Ultra-High Molecular Weight Polyethylene via Supercritical Nitrogen and Carbon Dioxide in Injection Molding

**DOI:** 10.3390/polym10010036

**Published:** 2017-12-30

**Authors:** Galip Yilmaz, Thomas Ellingham, Lih-Sheng Turng

**Affiliations:** 1 Polymer Engineering Center, Department of Mechanical Engineering, University of Wisconsin–Madison, Madison, WI 53706, USA; gyilmaz@wisc.edu (G.Y.); ellingham@wisc.edu (T.E.); 2 Wisconsin Institute for Discovery, University of Wisconsin-Madison, Madison, WI 53715, USA

**Keywords:** ultra-high molecular weight polyethylene (UHMWPE), microcellular injection molding, supercritical fluid, supercritical N_2_, supercritical CO_2_

## Abstract

The processability of injection molding ultra-high molecular weight polyethylene (UHMWPE) was improved by introducing supercritical nitrogen (scN_2_) or supercritical carbon dioxide (scCO_2_) into the polymer melt, which decreased its viscosity and injection pressure while reducing the risk of degradation. When using the special full-shot option of microcellular injection molding (MIM), it was found that the required injection pressure decreased by up to 30% and 35% when scCO_2_ and scN_2_ were used, respectively. The mechanical properties in terms of tensile strength, Young’s modulus, and elongation-at-break of the supercritical fluid (SCF)-loaded samples were examined. The thermal and rheological properties of regular and SCF-loaded samples were analyzed using differential scanning calorimetry (DSC) and parallel-plate rheometry, respectively. The results showed that the temperature dependence of UHMWPE was very low, suggesting that increasing the processing temperature is not a viable method for reducing injection pressure or improving processability. Moreover, the use of scN_2_ and scCO_2_ with UHMWPE and MIM retained the high molecular weight, and thus the mechanical properties, of the polymer, while regular injection molding led to signs of degradation.

## 1. Introduction

Ultra-high molecular weight polyethylene (UHMWPE) is a linear homopolymer polyethylene that possesses many desirable solid-state characteristics [1]. When comparing UHMWPE to well-known polyethylene grades such as high-density polyethylene (HDPE), its distinct properties are directly associated with its ultra-high molecular weight, which ranges from 1 million g/mol to 6 million g/mol, compared to 0.05–0.25 million g/mol for HDPE [2,3,4]. Owing to its ultra-high molecular weight, UHMWPE has many high-performance properties, including high abrasion resistance, high toughness and fiber modulus, great chemical inertness, low friction coefficient, self-lubrication behavior, very low service temperature, noise dampening, and more.

As a result, UHMWPE has found successful applications in many industrial and medical sectors. For example, it has been employed in high-performance fiber products, artificial joint prosthesis, bearings, bumpers, and the sliding parts of production lines [4,5,6]. Its chemical inertness, low friction, and wear-resistant features make it a useful material for parts where lubricants could cause contamination or maintenance problems, such as in food and medical applications [1,7]. 

However, the ultra-high molecular weight that yields these desirable properties and applications also hinder the fabrication of UHMWPE by standard polymer processing methods, such as injection molding (IM), as it requires a very high processing pressure due to its high melt viscosity and lack of fluidity [1,4,8].

In the plastics processing industry, injection molding is one of the most important processes due to its wide range of processable materials, ready-to-use final products, capability to make complex parts, short cycle time, and high degree of automation [9]. It is estimated that injection molding is used to process approximately one-third of all thermoplastic materials, with the process encompassing around one-half of all plastic processing equipment [10].

At present, only a few UHMWPE grades in granular form are available for injection molding. Its original powder form is unsuitable for injection molding, even when using specially designed screws for powder resins. Due to its high viscosity, molding UHMWPE requires high injection rates and injection pressures (values such as 110 MPa compared to 55–70 MPa for most polymers) [3,11]. Although the special grades of UHMWPE allow for injection molding, a further understanding of their behavior is necessary to take advantage of all of the potential benefits and achieve optimal and reliable processing conditions. Moreover, if the processing of UHMWPE is not performed properly, the product quality can suffer due to thermal degradation. 

Given its processing challenges, research literature on the injection molding of UHMWPE remains scarce. To the best of our knowledge, injection molding of UHMWPE is limited to Kuo’s work [12,13,14]. In these studies, the injection molding of UHMWPE, process optimization for improved mechanical properties, and tribological characteristics were investigated.

There are two well-known approaches for successfully fabricating UHMWPE parts: (1) using high-pressure settings or (2) reducing the viscosity via solvents [1]. Solvents, including mineral oil, paraffin wax, decalin, and xylene, have been used successfully to process UHMWPE [6]. However, some tedious post-processing operations are necessary to clean parts and reclaim and recycle the solvents. Also, the high pressures required for processing increases tool wear and manufacturing costs. 

An effective method for overcoming the high viscosity of UHMWPE is by using supercritical fluid (SCF) as a plasticizer. A compressed atmospheric gas, such as N_2_ or CO_2_ above its critical temperature and pressure values (cf. Table 1), is referred to as an SCF. An SCF exhibits the density of a liquid but has the high diffusivity and low viscosity of a gas. This specific phase allows not only accurate metering and pumping as a liquid, but also an increased diffusion rate into the polymer as a gas [15,16].

Once SCF is diffused into the polymer melt evenly, it can reduce the viscosity and the glass transition temperature of the polymer depending on the weight percentage of the SCF used [17,18]. The physical effect that SCF has on UHMWPE can be described by two mechanisms. The first and dominant mechanism is the increased free volume among entangled polymer chains [19,20]. The secondary mechanism is decreased chain entanglement due to the dilution of the polymer via the addition of the dissolved SCF [21]. The SCF also acts as a reversible plasticizer because it can leave the polymer matrix simply by diffusing out after processing is complete or when the thermodynamic instability is triggered due to changes in pressure or temperature. However, there are some drawbacks of SCFs, such as the need for high-pressure equipment. Nonetheless, SCF is still commonly used with favorable results because it can eliminate tedious solvent handling and all post-processing operations [22,23,24].

In this study, atmospheric gases, which are less expensive and unregulated—namely, N_2_ and CO_2_, were used in their supercritical state [15]. Several studies have been done on the plasticizing effects of supercritical CO_2_ in various polymer processing methods [21,23,24,25,26,27]. Wilding et al. showed that the effect of supercritical CO_2_ on viscosity intensifies as the molecular weight of the linear PE grade increases [28]. This suggests that the viscosity of UHMWPE can be reduced effectively via SCF. Moreover, supercritical N_2_ has received some attention as an alternative to CO_2_ for processing, especially for polyethylenes like low-density polyethylene (LDPE) [29]. In practice, injection molding using supercritical N_2_ as a foaming agent is more common because it yields a finer and denser foamed microstructure [22,30]. The main advantage of N_2_ over CO_2_ is that the back pressure required to keep N_2_ in its supercritical state is much lower (~54%) than the back pressure required for CO_2_ [16]. A higher back pressure requires a longer dosage time, which is undesirable. On the other hand, the estimated maximum gas solubility of CO_2_ is almost four times higher than N_2_ in PE at 200 °C [15]. Therefore, CO_2_ may have more potential to modify the melt properties when a higher amount of SCF is needed.

One of the commercially available technologies used to introduce SCF into the polymer is the microcellular injection molding (MIM) process. In this process, SCF is first dosed and injected into the injection molding barrel and mixed with the polymer melt during the dosage cycle. Thanks to the properties of SCFs, it diffuses to form small gas bubbles and eventually dissolves in the polymer matrix, thus creating a so-called “single-phase polymer/SCF solution” [15,16,29]. As the high pressure polymer/SCF solution enters the mold cavity, a sudden pressure drop causes the nucleation of bubbles, which expand to create a foamed product without the need for a packing cycle. This is because the expanding gas bubbles increase the volume of the foamed polymer, which compensates for material shrinkage as it cools, and thus fills the entire mold cavity [31,32,33]. The main usage of the MIM process is to create very fine and lightweight polymer foams with a typical average cell size of 3–100 μm [16,34] and a cell density of over 10^6^ cells/cm^3^. 

Occasionally, solid products instead of foamed ones can also be produced while utilizing SCF in the MIM process. This process option is called high-pressure foam injection molding (FIM) [35]. In this case, the entire mold cavity is filled with a full shot volume’s worth of material and then packing pressure is applied to suppress gas expansion and re-dissolve the gas into the polymer melt. In high-pressure FIM, the cells may or may not be eliminated completely depending on the processing parameters, such as the pressure in the cavity, SCF content, temperature, and polymer type [35,36].

In this study, the high-pressure foam injection molding option of the MIM process was used to produce solid parts rather than foamed ones in the UHMWPE injection molding experiments. Utilization of the plasticizing effect of supercritical N_2_ and CO_2_ was the aim. After the injection cycle, packing pressure was applied to block cell nucleation and re-solubilize the escaping gas. The injection molding of foamed UHMWPE parts using MIM was found to be a more involved project than their solid counterparts and remains an active on-going research subject. Herein, the processability in terms of injection pressure values and mold filling behavior of tensile bars, tensile properties, rheological properties, DSC data, and microstructure in forms of micro-computed tomography (μCT) images of the solid samples will be presented. 

## 2. Materials and Methods

An injection molding grade of UHMWPE resin (GUR^®^ 5129) was provided by Celanese Corporation (Irving, TX, USA). It had a viscosity average molecular weight of 4.7 million g/mol and a density of 0.93 g/cm^3^. Industrial grade carbon dioxide (CO_2_) and nitrogen (N_2_) were purchased from Airgas (Greenville, SC, USA) and used as the SCF source for MIM. 

### 2.1. Methods

A MIM-equipped injection molding machine, Arburg Allrounder 320S (Arburg, Lossburg, Germany) with a 25 mm diameter screw, was used to introduce scCO_2_ and scN_2_ into the melt. Here, the main purpose was to make a solid part rather than a foamed product by applying sufficient packing pressure to compress the full-size melt and suppress foaming. One critical requirement for the injection molding of UHMWPE is that the compression ratio of the screw must be less than 2.5:1, and preferably 2:1, due to the absence of melt flow based on the ISO 1133 standard. The compression ratio of the injection molding screw can be defined as the ratio of the feed section channel depth to the metering section channel depth [10]. The machine used in this study had a 2.5:1 compression ratio. Table 1 shows the injection-molded sample types and SCF loading percentages used in this study. The percent of SCF loading used was determined experimentally to introduce a high and controllable amount of SCF.

The processing parameters used for injection molding are listed in Table 2 and were set according to the material’s molding recommendations and the machine type used. The back pressure was adjusted for each gas to keep the SCF in its supercritical state. The cooling time was calculated as 25.2 s based on thermal diffusivity and target ejection temperature (85 °C) [37].

**Figure 1:**
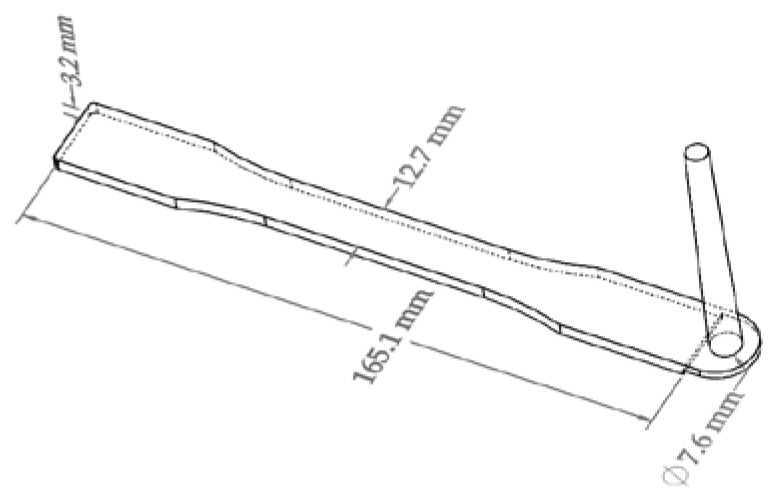
Schematic of the injection-molded specimen ASTM D638 Type I tensile bar mold cavity with its sprue.

An aluminum mold designed and machined in-house was used to produce ASTM standard Type I tensile bars with a 2 mm thick fan gate. Figure 1 shows the mold cavity and the sprue location, which was located on top of the fan gate without a runner to minimize additional pressure requirements.

Prior to any characterization experiments, the injection-molded UHMWPE samples were stored for at least one week to allow the dissolved gas to completely diffuse out.

### 2.2. Differential Scanning Calorimetry

Differential scanning calorimetry (DSC) was conducted according to the ASTM F2625-10 (2016) standard test method on a TA Instruments Q20 (TA Instruments, New Castle, DE, USA) to measure the enthalpy of fusion, percent crystallinity, and the melting point of the UHMWPE samples. The procedure was applied from ambient temperature to 200 °C, with a heat/cool/heat cycle at a 10 °C/min scanning rate. Four tests for each type of sample were performed with a weight range of 5 to 8 mg. Test samples were cut from the middle of the tensile bar. The enthalpy of melting per sample mass was normalized against the theoretical enthalpy of melting of 100% crystalline polyethylene to calculate the percentage of crystallinity in the test sample. The standard equation was used as follows:

(1) $$\%\;\mathrm{Crystallinity}=100\times \frac{\Delta H_{s}}{\Delta H_{f}}$$

where Δ*H*_s_ was the sample’s heat of fusion per unit mass during the freezing transition. The heat of fusion of 100% crystalline UHMWPE, Δ*H*_f_ = 289.3 J/g, was used in the calculation [38]. 

### 2.3. Rheology

A stress-controlled rheometer, TA Instruments AR 2000ex (TA Instruments, New Castle, DE, USA), was used to measure the complex viscosity of the samples. Tests were conducted with 25 mm diameter parallel plates with a 1 mm gap. Samples were cut from the tensile test bars, then compression molded at 200 °C for 10 s at 1 mm thickness. When room temperature was reached, the samples were punched into 25 mm diameter disks. First, a stress sweep test was run to determine the linear viscoelastic region and 0.5% strain was selected for the following tests. Frequency sweep tests were conducted from 0.01 Hz to 100 Hz at a temperature of 260 °C. For temperature dependency analyses, temperature ramp tests were run from 180 °C to 280 °C at two different frequencies, 0.1 and 100 Hz, respectively.

The temperature dependence of the materials was calculated using the following equation [39],

(2) $$m\left(T\right)=m_{o}\cdot exp\left[-a\left(T-T_{o}\right)\right]$$

where *m*(*T*) is the consistency index, *m*_o_ is the consistency index at the reference temperature *T*_o_, *a* is the temperature dependence or sensitivity coefficient, and *T* is the temperature at which the viscosity was measured. 

### 2.4. Micro-Computed Tomography (μCT)

To examine if any bubbles formed in the samples, μCT scans were conducted with an industrial type Metrotom 800 μCT system from Carl Zeiss AG (Oberkochen, Germany). The scanning power was set at 32 kV and 120 μA for a 5 μm spot and voxel size. Image processing was performed using ImageJ software from the National Institutes of Health, Bethesda, MD, USA.

### 2.5. Tensile Tests

Tensile testing of the samples was performed on an Instron 5967 (Instron, Norwood, MA, USA) with a 30 kN load cell using ASTM D638-03 Type I tensile specimens. At least five samples of each type were tested with a crosshead speed of 50 mm/min as recommended in the standard. Before testing, all samples were examined with transmitted light to check for any remaining bubbles. Samples with defects were excluded. Moreover, the samples were weighed and compared to each other to eliminate any odd samples. A few samples were one standard deviation away from the mean and were also excluded.

### 2.6. Tensile Bar Images and Injection Pressure Measurements

To examine the mold-filing behavior of UHMWPE melt, the injection volume was reduced by 20% and the holding stage was canceled so that the injection pressure reduction and the effect of dissolved SCF on the shape of the short shots could be observed. Pressure data were collected at least five times for each injection speed setting from 10 cm^3^/s to the maximum machine injection speed of 80 cm^3^/s in 10 cm^3^/s increments. All samples were weighed each time to ensure that the same amount of material was injected within 0.1 g. 

## 3. Results and Discussion

### 3.1. Differential Scanning Calorimetry

**Figure 2:**
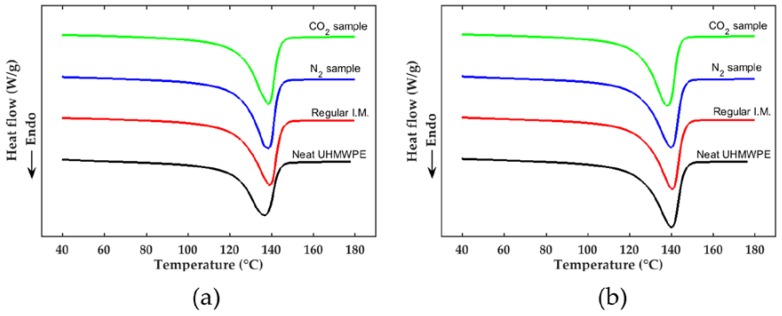
(**a**) The first heating and (**b**) the second heating of the differential scanning calorimetry (DSC) graphs of the injection-molded samples and neat ultra-high molecular weight polyethylene (UHMWPE).

The first and second heating DSC graphs of the neat and injection-molded (regular, CO_2_, and N_2_) samples can be seen in Figure 2, and the data derived from the DSC test is reported in Table 3 and Table 4 for the first and second heating tests, respectively. 

In Figure 2a, the first heating curves for all injection-molded samples seemed identical and showed a higher melting point than the neat sample. It is known that different thermomechanical (temperature–pressure) histories, as well as material degradation, can alter the first heating thermograms. The distinct factor in the thermal history of the injection-molded samples is expected to be the presence of scN_2_ and scCO_2_ during processing. However, the degree of crystallinity (% crystallinity) of the samples tabulated in Table 3 was similar within the standard deviation. The degree of crystallinity of the neat sample was much less than the injection-molded samples, likely due to a different thermomechanical history from its production process.

The difference between the regular and SCF-loaded injection-molded samples was smaller than the standard error for each of them, thus indicating that SCF did not have a clear effect on the thermal properties. Even so, the N_2_ and CO_2_ samples had slightly higher-than-average degrees of crystallinity as compared to the regular sample—1.0% more for the N_2_ samples and 0.4% more for the CO_2_ samples. This behavior might have been due to the slightly increased molecular mobility and higher packing pressure on the N_2_ and CO_2_ samples. As mentioned earlier, scN_2_ and scCO_2_ can reduce the viscosity of the polymer by increasing the free volume between polymer chains. Therefore, it is expected to enhance the crystallization kinetics and degree of crystallinity. In addition, lower flow resistance on the polymer should transfer more pressure during the packing cycle. On the other hand, the foaming and melt fracture mechanisms during the filling stage caused some gas to escape and vent out just before the packing stage. This lowered the amount of SCF in the melt during the cooling phase and the packing cycle, which decreased crystallization in these phases.

Since the crystal structure is very rigid compared to the amorphous structure, a higher degree of crystallinity usually increases the Young’s modulus of the final product [40]. As will be seen later, the Young’s modulus in the tensile tests in this study followed the results of the DSC tests. In the absence of SCF, the degree of crystallinity for the regular sample was slightly lower than for the SCF samples. Meanwhile, the average melting points were similar among the samples. 

Because the second heating had the same thermal history, the second heating thermograms should reflect the thermal degradation of the material rather than the processing history. It is known that a lower molecular weight PE, namely HDPE, tends to have a higher degree of crystallinity [41]. The data in Table 4 show that all injection-molded samples had slightly higher degrees of crystallinity than neat UHMWPE, although there were no significant differences in degrees of crystallinity or melting points among the injection-molded samples.

Yasuniwa et al. reported that, although the melting point of the PE grades increased linearly with the molecular weight, above 10^6^ g/mol, the change was insignificant [42]. The results in our study agree with this finding. While the melting points of all injection-molded samples were similar, the rheology and mechanical test results to be discussed below showed that the regular sample had a lower viscosity and tensile properties than the SCF samples.

One possible source of error in the DSC tests could stem from a non-uniform distribution of material with different molecular weights in the final product. Within the injection-molding barrel, thermal degradation mainly occurs on the surface of the barrel rather than on the screw root. Once the material enters the cavity, material degradation takes place where the temperature (due to viscous heating) or stress (due to shear and elongational flows) is highest. Although four tests for each type of sample were performed, the degraded material may not have been distributed and dispersed evenly within the main matrix.

### 3.2. Mechanical Properties

**Figure 3:**
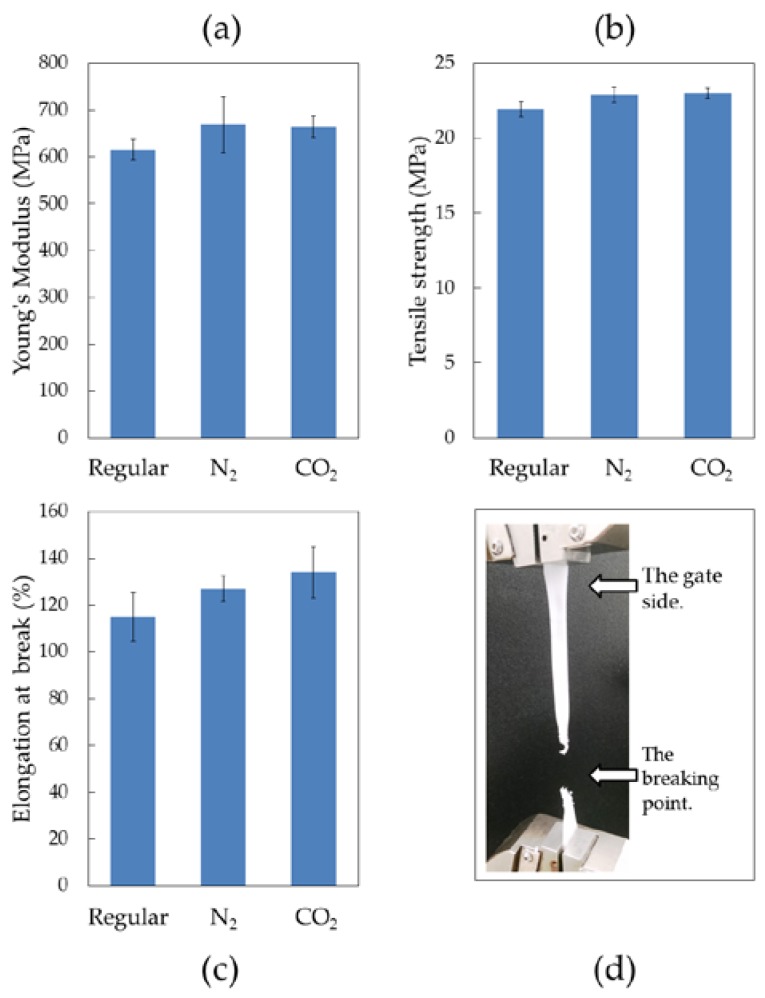
(**a**–**c**) Mechanical properties of UHMWPE samples and (**d**) an image of the breaking point.

The bar graphs in Figure 3a–c show the mechanical properties of the injection-molded samples. The same data are also tabulated in Table 5. The Young’s moduli of the N_2_ and CO_2_ samples increased slightly from 8% to 9% compared to the regular sample. A similar slight increase was also observed for tensile strength, with about 1 MPa of difference. 

The largest improvement was observed on the elongation-at-break. The N_2_ sample had a 12% elongation-at-break increase and the CO_2_ sample had a 19% elongation-at-break increase, indicating that the samples with super-critical gas deformed more before fracturing than the regular sample. 

During the mechanical tests, all parts ruptured in the same thin section on the side farthest from the gate (cf. Figure 3d). Elongation-at-break was mainly determined by the weakness of this point. This weakness was attributed to relatively low pressure at that point along the part during the packing cycle, as the pressure during the packing phase decreased from the gate to the end of the part. The dominant role of the melt pressure on the mechanical properties of injection-molded parts is a well-known phenomenon [43,44]. However, melt pressure values across the same part do not vary much for low viscosity materials. Here, the reason might be that the high viscosity of the UHMWPE caused a relatively high pressure variance, which resulted in weakness at the indicated far-end location on the tensile bar. While the N_2_ samples exhibited a slightly higher Young’s modulus, the CO_2_ samples had higher values in terms of elongation-at-break and toughness. However, the differences between the N_2_ and CO_2_ samples were within the standard variation. The reason for this might have been that both gases had similar physical effects on the flow behavior.

### 3.3. Rheology

**Figure 4:**
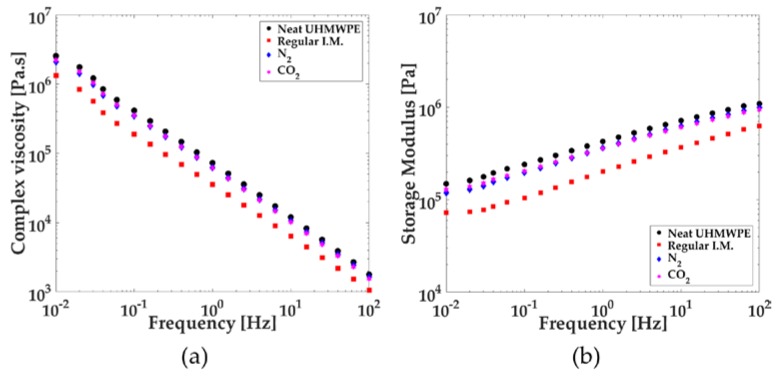
(**a**) Complex viscosity versus frequency; (**b**) Storage modulus versus frequency.

UHMWPE is normally selected as a material for its excellent impact strength and wear resistance based on its ultra-high molecular weight. This high molecular weight must be preserved to an acceptable level after thermal processing via a process like injection molding. The molecular weight of the final product should be checked to determine whether the process was successful or not. Due to the high molecular weight of UHMWPE and the difficulty of accessing a suitable solvent for the gel permeation chromatography (GPC) device, the molecular weight of the neat and injection-molded samples were analyzed in an indirect way by examining the change in melt viscosity. Moreover, a better understanding of the rheological characteristics of UHMWPE may help design processing parameters. Figure 4a displays the complex viscosity of neat and injection-molded UHMWPE samples.

In the tested frequency range in Figure 4a, all UHMWPE samples fit the Power-Law model with a strong shear-thinning behavior and without a zero-shear rate Newtonian viscosity plateau. At the lowest frequency tested, 0.01 Hz, the complex viscosity of neat UHMWPE corresponded to 2.553 × 10^6^ Pa·s. Regular injection-molded UHMWPE samples processed without any SCF had a complex viscosity of 1.324 × 10^6^ Pa·s, which was about 48% lower. On the other hand, the viscosities of the N_2_ and CO_2_ samples were closer to the neat sample, with an 18% reduction for N_2_ samples and a 12% reduction for CO_2_ samples. This viscosity reduction was likely due to material degradation as a result of viscous heating, especially during the recovery or dosage cycles and the injection-molding process. 

Thermal degradation lowers the molecular weight and broadens its distribution by breaking the polymer chains. Although increasing the amount of low-molecular-weight chains in the polymer typically improves flowability, it degrades the impact strength and wear resistance of the final products [39,45]. Based on our experimental observations, the thermal degradation of UHMWPE was minimized with proper machine and mold selection, such as using a smaller compression-ratio screw, small volume part, and a larger gate. Independent of this, the rheological results suggested that SCF helped minimize the thermal degradation of UHMWPE.

Figure 4b shows the storage moduli of neat and injection-molded UHMWPE samples. It can be seen that the storage moduli of the N_2_ and CO_2_ samples behaved similarly to the viscosity results, showing only a slight decrease compared to the neat sample, whereas the regular injection-molded sample displayed a comparatively large decrease. 

Equation (3) shows the expression of viscous heating upon shear deformation:

(3) $${\dot{Q}}_{\mathrm{VH}}=\eta {\dot{\gamma }}^{2}.$$

In the above equation, ${\dot{Q}}_{\mathrm{VH}}$ is the rate of viscous heating, η represents the shear viscosity, and $\dot{\gamma }$ is the shear deformation rate [39,45]. Thermal degradation of a material that occurs during processing is largely the result of the viscous heating. Although all samples except the neat sample were produced using the same processing parameters, the viscous heating might have been lower for the N_2_ and CO_2_ samples due to the plasticizing effect of the SCF, which lowers viscosity [17,46].

**Figure 5:**
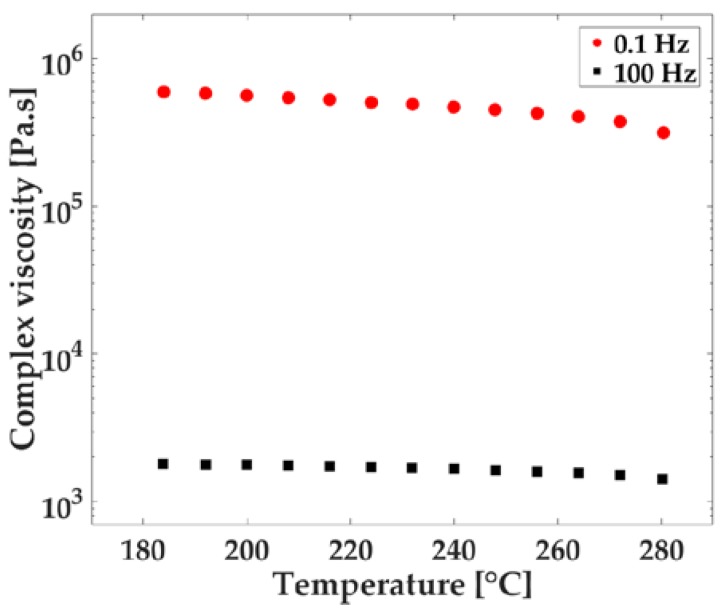
Complex viscosity versus temperature.

The temperature dependence of the UHMWPE viscosity should be understood for successful manufacturing. For this purpose, a temperature sweep test was performed and reported in Figure 5 at two frequencies (0.1 and 100 Hz). Only a small temperature dependence was observed for the UHMWPE viscosity, as shown by the slope of the data points in Figure 5 for both frequencies tested. The temperature sweep tests suggested that increasing the temperature of the UHMWPE melt for processing ease was not an effective way of decreasing the viscosity of the polymer, despite its common practice.

When temperature dependence, based on the temperature dependence relationship presented in Equation (2), was compared among common thermoplastics (HDPE, LDPE, Polystyrene (PS), Polypropylene (PP), Polyamide (PA66), Polycarbonate (PC), and Polyvinyl chloride (PVC)), from a general standpoint, the smallest temperature dependence coefficient, *a*, was reported as 0.002 °C^−1^ for HDPE [39]. According to the same model, the temperature dependence coefficient of UHMWPE was calculated as 0.001 °C^−1^, about half that of HDPE’s dependence. Low temperature dependence for a polymer can be a desirable property for a reliable injection-molding process. Therein, the polymer can maintain its viscosity within stable limits, despite factors like viscous heating or varying zone temperatures along the barrel. However, having a lower temperature dependence shifts the focus to the resistance to flow which, regardless of the melt temperature, becomes the major impediment in processing UHMWPE. 

### 3.4. Tensile Bar Images and Injection Pressure Measurements

**Figure 6:**
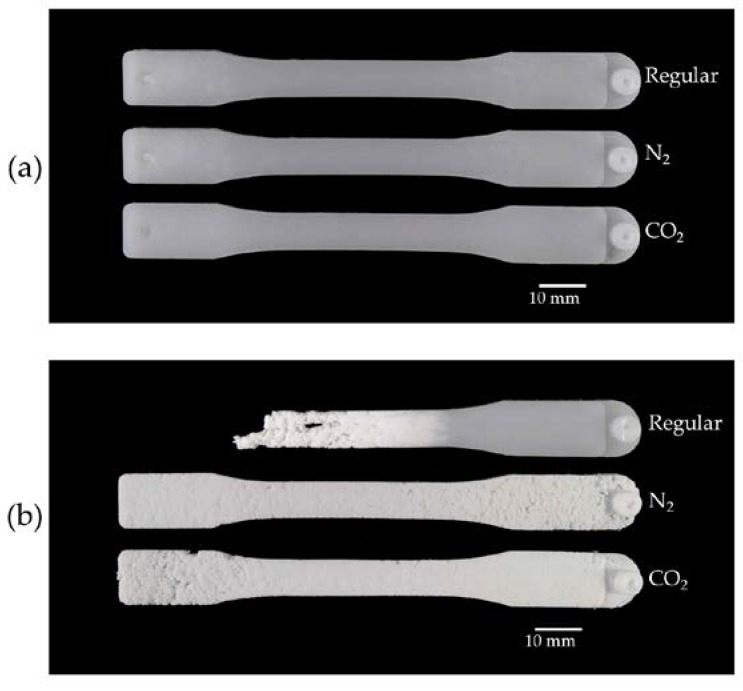
Images of injection-molded UHMWPE samples: (**a**) complete parts and (**b**) short-shot samples.

Images of complete and short-shot injection-molded UHMWPE parts are shown in Figure 6a,b. The gate location was on the right side of the parts. Although the appearance of the complete parts in Figure 6a was identical for all sample types, the short-shot images in Figure 6b showed a unique difference in flow behavior between the regular injection-molded samples and the SCF samples. It is well-known that many engineering plastics exhibit so-called “fountain flow” behavior [47]. However, in Figure 6b, it can be seen that the UHMWPE melt failed to demonstrate fountain flow behavior. This was because the material slipped at the mold surface as a result of its high viscosity and very low friction properties as well as the severe melt fracture that occurred as the melt entered the cavity under high stress. As a result, the flow front of the regular short-shot sample formed an irregular porous structure that became less porous near the gate. This might have been due to the huge pressure near the gate region where the part transitioned from a porous-like structure to a continuous-like structure. That is, the high-pressure development in the gate area caused the polymer to form a solid structure near the gate of the regular injection-molded part. Interestingly, it was observed that the N_2_ and CO_2_ samples in Figure 6b filled the entire cavity with a completely porous-like, foam-like structure, even near the gate side, as a result of gas expansion and venting. Such a porous structure was later compacted into solid-like parts (cf. Figure 6a) through packing pressure during the packing stage.

**Figure 7:**
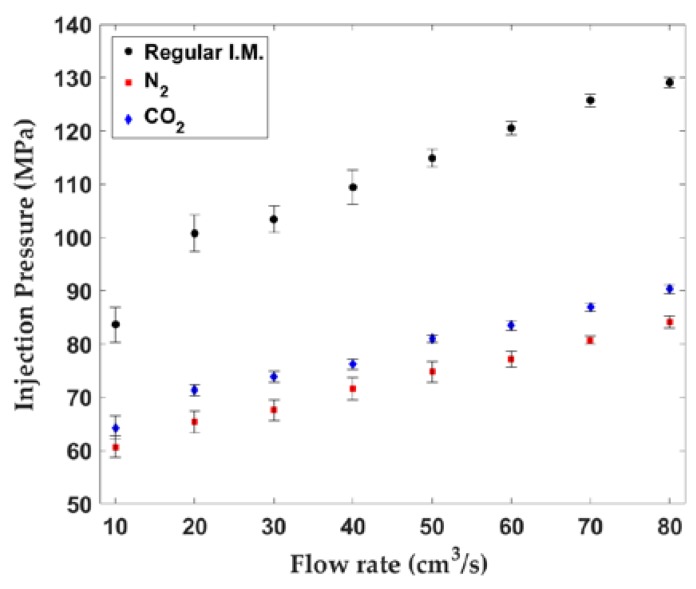
Injection pressure versus flow rate.

Figure 7 shows the injection pressure values versus flow rate on a linear scale axis. There was a noticeable decrease between the regular injection-molded and SCF-loaded samples. The N_2_ samples yielded slightly lower pressure values compared to the CO_2_ samples.

The shapes of the curves, especially from moderate to high flow rates, seemed linear. For the 80 cm^3^/s flow rate used to create the injection-molded samples, the average pressure reduction was about 30% for CO_2_ samples and 35% for the N_2_ samples.

This pressure reduction might have been due to the viscosity reduction effects of the dissolved SCF in the polymer melt. Previous studies have reported this effect for scN_2_ and scCO_2_ on various PE grades [28,29,46].

Other contributions to the pressure drop may have been that the material changed its flow behavior as demonstrated in Figure 6b. It seems that SCF decreased the material’s tendency to agglomerate and stick to the mold surface, thus resulting in less pressure development. Furthermore, the escaping high-pressure gas during the filling stage may have caused a lubrication effect and slipping between the UHMWPE part and the mold surface. 

Another reason for the pressure reduction might have been undissolved gas bubbles in the screw chamber or emerging gas bubbles in the mold cavity, which may also explain why scN_2_ reduced the pressure slightly more than scCO_2_. In microcellular injection molding (MIM), until the injection cycle begins, it is assumed that finely distributed micro-gas bubbles completely dissolve in the polymer melt and create a single-phase solution. However, in practice, the single-phase polymer/gas solution may not form completely at certain molding settings over a typical recovery time [16]. As long as the back pressure is kept above the super critical pressure, the residual micro-bubbles will not affect the MIM process [16]. 

The gas solubility in polymers is a strong function of pressure and temperature. Based on our experimental observations, it was estimated that the residual microbubbles when scN_2_ was used were more numerous than with the scCO_2_ case because of the lower solubility of scN_2_ in the UHMWPE melt. To the best of our knowledge, gas solubility and diffusion data are not available for UHMWPE. For one grade of HDPE, the gas solubility was reported as 1.2 wt % for scN_2_ and 4.6 wt % for scCO_2_ at 270 °C [16]. Similarly, as gas emerges from the polymer/gas solution during injection, numerous bubbles form due to thermodynamic instability. Since scN_2_ has a lower solubility in UHMWPE, it will have a higher degree of supersaturation for scN_2_ given the same gas loading at 1.5 wt % (cf. Table 1). Thus, more gas bubbles are expected to form for the N_2_ samples. Either way, tiny bubbles with relatively negligible viscosity may reduce the average flow resistivity of the melt-gas mixture.

### 3.5. Micro-Computed Tomography (μCT)

**Figure 8:**
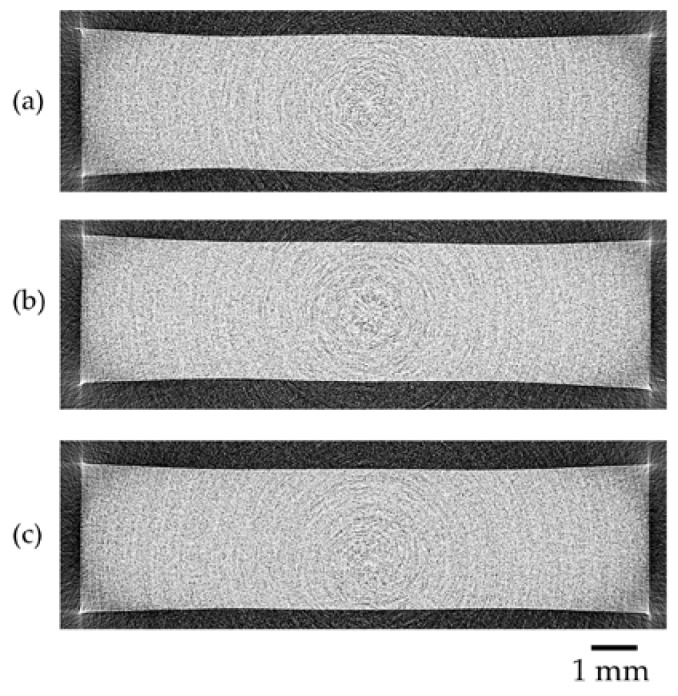
Micro-Computed Tomography (μCT) images of injection-molded samples: (**a**) regular injection-molded sample; (**b**) N_2_ sample; and (**c**) CO_2_ sample.

Although μCT resolution (5 μm) is limited when compared to electron microscopy methods, as a nondestructive method, it enables us to scan large volumes conveniently instead of only a limited area of the fracture surface. It also allows one to effectively analyze and select the most representative cross-section images. μCT images of injection-molded samples are shown in Figure 8. 

Remaining bubbles in injection-molded parts can cause some cosmetic and mechanical defects. When SCF is used, remaining bubbles can be a challenge to eliminate. During cooling, the shrinkage of the polymer can create some random bubbles in that part. However, the injection molding images in Figure 6b show that UHMWPE underwent foaming and allowed gas to emerge and escape. Even if there is a relatively small amount of gas remaining, it is easy to pack the part and eliminate the bubbles for both N_2_ and CO_2_ samples, resulting in solid injection-molded parts free of any residual bubbles as shown in Figure 8. In μCT images of all injection-molded samples, the corners of the cross sections images demonstrated unexpectedly sharp and light colored edges. This might have been due to the exponential edge-gradient effect observed as a defect of the μCT mathematical reconstruction algorithm whenever long sharp edges of high contrast are encountered [48]. 

## 4. Conclusions

In summary, UHMWPE was processed via microcellular injection molding with supercritical N_2_ or CO_2_ as a reversible plasticizer and compared with regular injection-molded samples. It was found that both scN_2_ and scCO_2_ reduced the required injection pressure for the chosen material flowrate during injection. The flow behavior was also found to be different when scN_2_ and scCO_2_ were used. UHMWPE failed to demonstrate typical fountain flow behavior; instead, it slipped on the mold surface. While DSC results showed a small difference in terms of the degree of crystallization and melting temperature among all samples, the rheological tests showed that the complex viscosity and storage modulus of the N_2_ and CO_2_ samples were closer to that of the neat sample and higher than the regular injection-molded sample. This suggests that some thermal degradation may have occurred during processing, but scN_2_ and scCO_2_ helped reduce the overall degradation due to the plasticizing effect in the barrel and injection molding cycle. In addition, the rheological tests showed that increasing the melt temperature would not reduce the viscosity. From the mechanical tests, it was found that CO_2_ samples had the most favorable elongation-at-break results while N_2_ samples had the highest Young’s modulus. Finally, μCT images showed that N_2_ and CO_2_ samples were free of any residual bubbles.

## Figures and Tables

**Table 1:** Sample name and SCF loading percent.

Sample Name	SCF	Critical Points [15]	Loading % by Weight
Regular	N/A	N/A	0
CO_2_ sample	CO_2_	31 °C, 7.37 MPa	1.5
N_2_ sample	N_2_	−147 °C, 3.40 MPa	1.5

**Table 2:** Processing parameters used in injection molding.

Processing Parameters	Units	Value
Injection speed	cm^3^/s	80
Injection vol.	cm^3^	17.2
Cooling time	s	25
Back pressure	MPa	Regular: 0.5 N_2_: 5 CO_2_: 8
Packing pressure	MPa	110
Packing time	s	7
Nozzle temperature	°C	260
Mold temperature	°C	80

**Table 3:** The average thermal behavior of injection-molded samples obtained from the first heating thermograms.

Sample	*T*_m_ (°C)	∆*H*_s_	% Crystallinity
Neat	136.9 ± 0.1	142.4 ± 1.9	49.2 ± 0.7
Regular	139.5 ± 0.4	158.2 ± 3.2	54.7 ± 1.1
N_2_ sample	138.7 ± 1.0	161.1 ± 2.7	55.7 ± 0.8
CO_2_ sample	138.6 ± 0.7	159.6 ± 2.2	55.1 ± 0.9

**Table 4:** The average thermal behavior of injection-molded samples obtained from the second heating thermograms.

Sample	*T*_m_ (°C)	∆*H*_s_	% Crystallinity
Neat	139.8 ± 0.6	166.7 ± 0.7	57.6 ± 0.2
Regular	140.3 ± 0.9	167.7 ± 2.7	58.0 ± 0.8
N_2_ sample	140.2 ± 0.8	169.8 ± 1.5	58.7 ± 0.5
CO_2_ sample	139.7 ± 1.2	167.6 ± 1.9	57.9 ± 0.6

**Table 5:** Tensile properties of injection-molded samples.

Samples	Young’s Modulus (MPa)	Elongation at Break (%)	Tensile Strength (MPa)	Toughness (J/m^3^)
Regular	615.2 ± 21.4	115 ± 10	21.9 ± 0.5	64.4 ± 7.1
N_2_ samples	669.6 ± 59.9	127 ± 5	22.9 ± 0.5	73.2 ± 4.0
CO_2_ samples	664.0 ± 22.7	134 ± 11	23.0 ± 0.3	77.8 ± 8.4

## References

[1] Peacock A.J. (2000). Handbook of Polyethylene: Structures, Properties and Applications.

[2] Edidin A.A., Kurtz S.M. (2000). Influence of mechanical behavior on the wear of 4 clinically relevant polymeric biomaterials in a hip simulator. J. Arthroplast..

[3] Osswald T.A., Baur E., Brinkmann S., Oberbach K., Schmachtenberg E. (2006). International Plastics Handbook.

[4] Kurtz S.M. (2009). The UHMWPE Biomaterials Handbook: Ultra-High Molecular Weight Polyethylene in Total Joint Replacement and Medical Devices.

[5] McKellop H., Shen F., Lu B., Campbell P., Salovey R. (1999). Development of an extremely wear-resistant ultra high molecular weight polythylene for total hip replacements. J. Orthop. Res..

[6] Schaller R., Feldman K., Smith P., Tervoort T.A. (2015). High-Performance Polyethylene Fibers “Al Dente”: Improved Gel-Spinning of Ultrahigh Molecular Weight Polyethylene Using Vegetable Oils. Macromolecules.

[7] Mourad A.-H.I., Fouad H., Elleithy R. (2009). Impact of some environmental conditions on the tensile, creep-recovery, relaxation, melting and crystallinity behaviour of UHMWPE-GUR 410-medical grade. Mater. Des..

[8] Xu M.M., Huang G.Y., Feng S.S., McShane G.J., Stronge W.J. (2016). Static and dynamic properties of semi-crystalline polyethylene. Polymers.

[9] Heim H.-P. (2015). Specialized Injection Molding Techniques.

[10] Osswald T.A., Turng L.-S., Gramann P.J. (2008). Injection Molding Handbook.

[11] Bryce B.D.M. (1996). Plastic Injection Molding Manufacturing Process Fundamentals.

[12] Kuo H.C., Jeng M.C. (2011). The influence of injection molding and injection compression molding on ultra-high molecular weight polyethylene polymer microfabrication. Int. Polym. Process..

[13] Kuo H.-C., Jeng M.-C. (2010). The influence of injection molding on tribological characteristics of ultra-high molecular weight polyethylene under dry sliding. Wear.

[14] Kuo H.-C., Jeng M.-C. (2010). Effects of part geometry and injection molding conditions on the tensile properties of ultra-high molecular weight polyethylene polymer. Mater. Des..

[15] Okamoto K.T. (2003). Microcellular Processing.

[16] Xu J. (2010). Microcellular Injection Molding.

[17] Kikic I. (2009). Polymer-supercritical fluid interactions. J. Supercrit. Fluids.

[18] Hernández-Ortiz J.C., Van Steenberge P.H.M., Reyniers M.F., Marin G.B., D’hooge D.R., Duchateau J.N.E., Remerie K., Toloza C., Vaz A.L., Schreurs F. (2017). Modeling the reaction event history and microstructure of individual macrospecies in postpolymerization modification. AIChE J..

[19] Vrentas J.S., Duda J.L. (1977). Diffusion in Polymer-Solvent Systems. I. Reexamination of the Free-Volume Theory. J. Polym. Sci. Polym. Phys. Ed..

[20] D’Hooge D.R., Van Steenberge P.H.M., Reyniers M.F., Marin G.B. (2016). The strength of multi-scale modeling to unveil the complexity of radical polymerization. Prog. Polym. Sci..

[21] Nalawade S.P., Picchioni F., Janssen L.P.B.M. (2006). Supercritical carbon dioxide as a green solvent for processing polymer melts: Processing aspects and applications. Prog. Polym. Sci..

[22] Sun X., Turng L.S. (2014). Novel injection molding foaming approaches using gas-laden pellets with N_2_, CO_2_, and N_2_ + CO_2_ as the blowing agents. Polym. Eng. Sci..

[23] Tomasko D.L., Burley A., Feng L., Yeh S.-K., Miyazono K., Nirmal-Kumar S., Kusaka I., Koelling K. (2009). Development of CO_2_ for polymer foam applications. J. Supercrit. Fluids.

[24] Wingert M.J., Shen J., Davis P.M., Lee L.J., Tomasko D.L., Koelling K.W. Rheological Studies of Polymers under High Pressure Carbon Dioxide. Proceedings of the Society of Plastics Engineers Annual Technical Conference (ANTEC).

[25] Garcia-Leiner M., Song J., Lesser A.J. (2003). Drawing of ultrahigh molecular weight polyethylene fibers in the presence of supercritical carbon dioxide. J. Polym. Sci. Part B Polym. Phys..

[26] Kiran E. (2016). Supercritical fluids and polymers—The year in review—2014. J. Supercrit. Fluids.

[27] Ellingham T., Duddleston L., Turng L.-S. (2017). Sub-critical gas-assisted processing using CO_2_ foaming to enhance the exfoliation of graphene in polypropylene + graphene nanocomposites. Polymer.

[28] Wilding M.D., Baird D.G., Eberle A.P.R. (2008). Melt Processability and Foam Suppression of High Molecular Weight Polyethylenes Plasticized with Supercritical Carbon Dioxide. Int. Polym. Process..

[29] Hsu C.-L., Turng L.-S., Osswald T.A., Rudolph N., Dougherty E., Gorton P. (2012). Effects of Pressure and Supercritical Fluid on Melt Viscosity of LDPE in Conventional and Microcellular Injection Molding. Int. Polym. Process..

[30] Sun X., Kharbas H., Peng J., Turng L.S. (2015). A novel method of producing lightweight microcellular injection molded parts with improved ductility and toughness. Polymer.

[31] Kharbas H.A. (2003). Developments in Microcellular Injection Molding Technology.

[32] Turng L.-S., Kharbas H. (2004). Development of a Hybrid Solid-Microcellular Co-injection Molding Process. Int. Polym. Process..

[33] Kharbas H., Ellingham T., Turng L.-S. Use of core retraction to achieve low density foams in microcellular injection molded polypropylene parts. Proceedings of the Technical Conference & Exhibition.

[34] Gong S., Yuan M., Chandra A., Kharbas H., Osorio A., Turng L.S. (2005). Microcellular Injection Molding. Int. Polym. Process..

[35] Shaayegan V., Wang G., Park C.B. (2016). Effect of foam processing parameters on bubble nucleation and growth dynamics in high-pressure foam injection molding. Chem. Eng. Sci..

[36] Shaayegan V., Wang C., Costa F., Han S., Park C.B. (2017). Effect of the melt compressibility and the pressure drop rate on the cell-nucleation behavior in foam injection molding with mold opening. Eur. Polym. J..

[37] Liang J.Z., Ness J.N. (1996). The calculation of cooling time in injection moulding. J. Mater. Process. Technol..

[38] Wunderlich B., Cormier C.M. (1967). Heat of fusion of polyethylene. J. Polym. Sci. Part A Polym. Phys..

[39] Osswald T.A., Rudolph N. (2015). Polymer Rheology: Fundamentals and Applications.

[40] Pang W., Ni Z., Chen G., Huang G., Huang H., Zhao Y. (2015). Mechanical and thermal properties of graphene oxide/ultrahigh molecular weight polyethylene nanocomposites. RSC Adv..

[41] Ergoz E., Fatou J.G., Mandelkern L. (1972). Molecular Weight Dependence of the Crystallization Kinetics of Linear Polyethylene. I. Experimental Results. Macromolecules.

[42] Yasuniwa M., Tsubakihara S., Nakafuku C. (1988). Molecular Weight Effect on the High Pressure Crystallization of Polyethylene. Polym. J..

[43] Kubáut J., Månson J.-A., Rigdahl M. (1983). Influence of mold design on the mechanical properties of high-pressure injection—molded polyethylene. Polym. Eng. Sci..

[44] Kalay G., Sousa R.A., Reis R.L., Cunha A.M., Bevis M.J. (1999). The enhancement of the mechanical properties of a high-density polyethylene. J. Appl. Polym. Sci..

[45] Osswald T.A., Menges G. (2012). Material Science of Polymers for Engineers.

[46] Sauceau M., Fages J., Common A., Nikitine C., Rodier E. (2011). New challenges in polymer foaming: A review of extrusion processes assisted by supercritical carbon dioxide. Prog. Polym. Sci..

[47] Beaumont J.P. (2004). Runner and Gating Design Handbook: Tools for Successful Injection Molding.

[48] Joseph P.M., Spital R.D. (1981). The exponential edge-gradient effect in X-ray computed tomography. Phys. Med. Biol..

